# Trainee-Led Informatics Leadership and Electronic Medical Record Documentation Compliance in a Psychiatry Residency Program

**DOI:** 10.1192/bjo.2026.11531

**Published:** 2026-06-30

**Authors:** Sazgar Hamad, Mohamad Makki, Yousaf Iqbal, Islam Mohamed, Ahmad Alater

**Affiliations:** Hamad Medical Corporation, Doha, Qatar

## Abstract

**Aims:**

High-quality clinical documentation is essential for patient safety, continuity of care, and compliance with regulatory standards. However, maintaining documentation quality within complex electronic medical record (EMR) systems can be challenging in training environments, particularly in psychiatry where documentation requirements are extensive. While resident involvement in quality improvement is encouraged, there is limited evaluation of structured trainee-led governance models in clinical informatics. This study aimed to evaluate the impact of a trainee-led informatics leadership initiative on EMR documentation compliance within the Psychiatry Residency Program at Hamad Medical Corporation (HMC). It was hypothesised that formal trainee leadership and continuous engagement would be associated with improved compliance across key documentation indicators.

**Methods:**

A longitudinal quality improvement evaluation was conducted following the establishment of the Cerner Documentation Leadership Subcommittee in August 2022. The subcommittee consisted of psychiatry residents assigned formal leadership roles focused on documentation quality and clinical informatics. The initiative included monthly educational sessions, targeted feedback on documentation performance, iterative workflow optimisation, and continuous auditing of documentation standards. Compliance was assessed using 16 documentation indicators aligned with Joint Commission International (JCI) standards. Longitudinal compliance data were extracted from Cerner audit reports and reviewed across 2023 to examine trends, improvements, and variability in documentation performance over time.

**Results:**

Following implementation of the trainee-led leadership model, sustained improvements were observed across multiple documentation domains. History and Physical documentation and Medication Reconciliation maintained consistently high compliance rates throughout the evaluation period, with average compliance ranging between 86% and 90%. Comprehensive Assessment documentation showed the most notable improvement, increasing from a baseline compliance rate of 13% to 77% by the end of the evaluation year. Active trainee involvement in institutional reaccreditation processes and collaboration with Health Informatics teams supported optimisation of Cerner documentation templates and improvements in clinical workflow. Periodic declines in selected metrics were observed during resident rotation changes and onboarding of new trainees, indicating the influence of workforce turnover and the need for ongoing education and reinforcement.

**Conclusion:**

This evaluation demonstrates that a structured trainee-led informatics leadership model can improve EMR documentation compliance within a psychiatry residency program. Embedding residents in formal documentation governance roles supported sustained quality improvement while promoting accountability, leadership development, and systems-based practice. Ongoing education and continuous monitoring are necessary to maintain gains, particularly during trainee transitions. Integrating informatics leadership into residency training may strengthen regulatory readiness and support safe, high-quality psychiatric care.

